# A Formalization of the Smith Normal Form in Higher-Order Logic

**DOI:** 10.1007/s10817-022-09631-5

**Published:** 2022-05-26

**Authors:** Jose Divasón, René Thiemann

**Affiliations:** 1 University of La Rioja, Logroño, Spain; 2 University of Innsbruck, Innsbruck, Austria

**Keywords:** Theorem proving, Isabelle/HOL, Local type definitions, Elementary divisor rings

## Abstract

This work presents formal correctness proofs in Isabelle/HOL of algorithms to transform a matrix into Smith normal form, a canonical matrix form, in a general setting: the algorithms are written in an abstract form and parameterized by very few simple operations. We formally show their soundness provided the operations exist and satisfy some conditions, which always hold on Euclidean domains. We also provide a formal proof on some results about the generality of such algorithms as well as the uniqueness of the Smith normal form. Since Isabelle/HOL does not feature dependent types, the development is carried out by switching conveniently between two different existing libraries by means of the lifting and transfer package and the use of local type definitions, a sound extension to HOL.

## Introduction

The Smith normal form (SNF) is a well-known canonical form of matrices [51]. It is commonly defined for matrices whose coefficients belong to a *principal ideal domain* (from here on, PID). A matrix is in SNF if its diagonal elements $\alpha _i$ satisfy $\alpha _i\ |\ \alpha _{i+1}$ (note that every number divides 0 and 0 only divides itself) and the rest of the elements of the matrix are zeros. Over a PID, there exists an algorithm to transform a matrix *A* into its corresponding Smith normal form *S* by means of invertible matrices, i.e., there exist invertible matrices *P* and *Q* such that $S = P\!AQ$. The Smith normal form plays an important role in different fields, such as control theory [42], combinatorics [50] and structure of lattice rules [41]. Another important application arises in algebraic topology, since it can be used to compute the homology of a chain complex. More generally, it is useful to compute persistent homology, which can be applied to process big volume of data [48] and to the analysis of digital images [8].

In this work, we present a formalization of several facts related to the SNF of a matrix in Isabelle/HOL. We present two algorithms (one is specific to transform a diagonal matrix into Smith normal form, the other one works for arbitrary matrices), together with some theorems on the underlying structures and uniqueness of the transformation. A very important part of this contribution is how it has been obtained in a context where submatrices play an important role and the underlying logic (HOL) does not feature dependent types. Throughout the paper we present the work that we have carried out involving two matrix representations, the one presented in the HOL Analysis library (where matrices are encoded as functions over finite types) and another one by Thiemann and Yamada [53], where a matrix is modeled by a function that maps natural numbers (the indices) to elements, together with two natural numbers that correspond to its dimensions. Depending on the algorithm that we want to verify or the theorem that we wish to prove, we carry out the formal proof in one of the representations (either because it is easier or simply impossible in the other representation), and later we move the results (maybe including intermediate steps or sometimes just the final result) to the other one. To this end, we crucially rely on the lifting and transfer package [33] and above all on *local type definitions*, a sound extension of HOL [39]. Thus, our work provides an interesting case study of how to use these novel Isabelle/HOL tools to overcome the expressivity problem that HOL has no dependent types. This approach was already carried out in a similar context [22], but the new connection goes one step further and permits reusing more results and combining algorithms of different libraries, which were not possible before.

The outline of the paper is as follows. In Sect. 2, we introduce the ring structures that we formalized and also present a brief introduction to Isabelle. The Isabelle matrix libraries and the required infrastructure are also explained there. In the following sections we show the main contributions of this paper:

- In Sect. 3, we present the methodology and the enhanced connection between two matrix representations and we also show a guided example on how to move theorems from one library to the other one.
- In Sect. 4, we show a verified algorithm to compute a SNF of a diagonal matrix. The algorithm is defined over Bézout rings (instead over PIDs). We also show that any diagonal matrix can always be transformed into SNF if and only if its entries belong to a Bézout ring.
- In Sect. 5, we present both a verified algorithm to transform arbitrary matrices into SNFs and necessary and sufficient conditions, in terms of the underlying ring, for the existence of SNFs for arbitrary matrices. Concretely, our algorithm works over elementary divisor rings, and that is precisely the structure in which any matrix can be transformed into a SNF. Some characterizations of elementary divisor rings and Hermite rings are also formalized.
- The certification approach to compute a SNF, based on external oracles, is formalized in Sect. 6.
- The SNF of a matrix over a GCD domain (a more general structure than PID) is unique up to multiplication by units. We formalize this result in Sect. 7.

We then present an overview of related work in Sect. 8. Finally, Sect. 9 shows the conclusions. The formalization is publicly available in the Archive of Formal Proofs [17] and comprises over 14,000 lines of code.

## Preliminaries

### Algebraic Structures and Definitions

We expect the reader to be familiar with ring theory and linear algebra, and we refer to introductory books [12, 52] for their standard notions. We clarify that by ring we mean commutative ring with unit. We also recall two important types of rings that are intensively used throughout the paper:

#### Definition 1

(*GCD ring*) A *GCD ring* ${\mathcal {R}}$ is a ring where every pair of elements has a greatest common divisor, i.e., there exists $\gcd (a,b)\in {\mathcal {R}}$ such that:

- $$\gcd (a,b) \mid a$$ and $$\gcd (a,b) \mid b$$
- For all $g \in {\mathcal {R}}$ such that $g\mid a$ and $g\mid b$, then $g \mid \gcd (a,b)$

The $\gcd$ operation could be neither unique nor computable. If the ring is an integral domain (it has no zero divisors), the structure is called GCD domain.

#### Definition 2

(*Bézout ring*) A *Bézout ring* ${\mathcal {R}}$ is a ring where the Bézout identity holds, i.e., for $a,b \in {\mathcal {R}}$ there exist $p,q,d \in {\mathcal {R}}$ such that $pa + qb = d$ and *d* is a greatest common divisor of *a* and *b*. If the ring is an integral domain, the structure is called Bézout domain.

We recall the following chain of inclusions of rings (analogous for integral domains):The previous definitions were already part of the Isabelle/HOL library. During the formalization process, we also need to introduce more algebraic structures. The following definition is by Gillman and Henriksen [26].

#### Definition 3

(*Admits triangular reduction*) An arbitrary matrix *A* over a ring ${\mathcal {R}}$ admits triangular reduction if there exists an invertible matrix *U* over ${\mathcal {R}}$ such that *AU* is lower triangular.

Let us remark that, by symmetry, we could have defined the same concept imposing the matrix $U\!A$ to be upper triangular. Now, we introduce the concept of an Hermite ring following the definition by Kaplansky [37].

#### Definition 4

(*Hermite ring*) A ring ${\mathcal {R}}$ is called an Hermite ring if every matrix *A* over ${\mathcal {R}}$ admits triangular reduction.

For the sake of completeness, we show the definition of the Smith normal form.

#### Definition 5

(*Smith normal form*) An arbitrary matrix *A* over a ring ${\mathcal {R}}$ is said to be in Smith normal form (abbreviated *SNF*), if it is a diagonal matrix and every diagonal element $A_{i,i}$ divides $A_{i+1,i+1}$.

#### Definition 6

(*SNF of a matrix*) A matrix *S* is said to be a SNF of an arbitrary matrix *A* over a ring ${\mathcal {R}}$, if *S* is in SNF and there exist invertible matrices (over ${\mathcal {R}}$) *P*, *Q* such that $P\!AQ = S$.

It is worth noting that the SNF is sometimes defined over a PID or a Euclidean domain in the literature, since it is possible to define an algorithm in such structures to compute the SNF of a matrix. However, as presented above, the SNF can be defined for matrices over any ring ${\mathcal {R}}$. Let us also note that by $A_{i,i} \ |\ A_{i+1,i+1}$ we mean that there exists an element $u \in {\mathcal {R}}$ such that $A_{i+1,i+1} = u A_{i,i}$, but neither a witness nor an executable division algorithm is required. A closely related concept is the definition of admissibility of diagonal reduction presented by Kaplansky [37]:

#### Definition 7

(*Admits diagonal reduction*) An arbitrary matrix *A* over a ring ${\mathcal {R}}$ admits diagonal reduction if there exist invertible matrices (over ${\mathcal {R}}$) *P*, *Q* such that $P\!AQ$ is in SNF.

Finally, we present the definition of elementary divisor rings, again following the one by Kaplansky [37]:

#### Definition 8

(*Elementary divisor ring*) A ring ${\mathcal {R}}$ is called an elementary divisor ring if every matrix *A* over ${\mathcal {R}}$ admits diagonal reduction.

### A Brief Introduction to Isabelle

Isabelle is a generic theorem prover which supports different object logics [45]. This is possible thanks to Isabelle’s logical framework Isabelle/Pure, a meta-logic that allows the formalization of the syntax and inference rules of a broad range of object logics. The most widespread object logic supported by Isabelle is higher-order logic (or briefly, HOL). Isabelle’s version of HOL (usually called Isabelle/HOL) corresponds to Church’s simple type theory [14] extended with polymorphism, Haskell-style type classes and type definitions. It is the one where the greatest number of tools (code generation, automatic proof procedures) and libraries are available. For instance, almost all the developments published in the Archive of Formal Proofs [5] (more than 600 articles) use HOL as their object logic.

Our formalization is based on Isabelle/HOL and we follow its syntax conventions throughout the paper. For instance, $f \,{:}{:}\, \alpha \Rightarrow \beta$ indicates that *f* is a function that maps elements of type $\alpha$ to elements of type $\beta$. Type classes [28] allow sharing operations and assumptions among different types (also notation and names). One can build hierarchies of abstract structures with them, but they include a considerable restriction: type classes are restricted to one type parameter. Despite this limitation, they have proved very useful in many developments and there are more than 180 type classes in the Isabelle/HOL standard library. They can be understood as a mechanism to impose additional restrictions over type variables; for instance, the expression  imposes the constraint that the type variable $\alpha$ possesses the structure and properties stated in the  type class, and can be later replaced exclusively by types which are instances of that type class. A class that is very important for our development is the  class, which assumes the type to have finitely many elements. 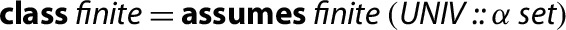


We use  in some Isabelle statements. It is a proposition which guarantees that the type $\alpha$ satisfies the required properties to be an instance of the type class *T*.

Locales [6] are a concept related to type classes. A locale fixes operations (or parameters) and assumptions over them, and provides a context in which theorems (implied by the fixed operations and their assumptions) can be proved. Locales are hierarchic and their theorems can be inherited in other contexts by means of interpretations. They are especially useful for abstract algebra developments [7], since they permit working explicitly with carrier sets, describe homomorphisms between structures, and they have no limitation on type variables.

The lifting and transfer package [33] allows one to transport definitions and theorems from one type to a related one. Since its introduction in 2013, this package has become a very useful tool in numerous different developments. Local type definitions were introduced by Kunĉar and Popescu as part of Isabelle/HOL in 2016. They provide a new rule for type definitions that extends the HOL logic. This rule brings some of the dependent type expressiveness into Isabelle/HOL, by emulating type definitions locally. Kunĉar and Popescu also proved the consistency of this new rule with the logic of Isabelle/HOL. The rule is formally written as:

LT $$\begin{aligned} \frac{\varGamma \vdash A \ne \emptyset \ \ \ \ \varGamma \vdash (\exists \mathrm{Abs}\ \mathrm{Rep}._{\ \sigma }(\beta \approx A)^\mathrm{Abs}_\mathrm{Rep})\rightarrow \varphi }{\varGamma \vdash \varphi }, \end{aligned}$$

where $\varGamma$ is a proof context, $\varphi$ is a formula, *A* is a nonempty set (of type $\sigma$), and Rep and Abs the functions which define an isomorphism between the set of all elements of a new type $\beta$ and *A*. Essentially, this rule allows assuming (locally) the existence of a type $\beta$ (with no type class restrictions) which is isomorphic to an arbitrary nonempty set *A*, where $\beta$ must be fresh. With some work, this permits going from types to sets (and to terms) in Isabelle/HOL. There are more technical details about this approach such as class internalization and the unoverloading rule. We refer to Kunĉar and Popescu’s article for further details [39].

Throughout the paper, Isabelle keywords are written in .

### The HOL Analysis Library and Its Matrix Representation

The HOL Analysis (or *HA* for short) library [36] is a set of Isabelle/HOL theories based on Harrison’s work in HOL Light [29] and contains theoretical results in mathematical fields such as analysis, topology and linear algebra. It is a huge library with more than 9000 lemmas and 400 definitions.

In the HA library, matrices are represented by means of *Harrison’s trick*: finite types encode natural numbers by means of their cardinality. Then, matrices with elements of type $\alpha$ are essentially represented as functions whose domain are elements over finite types, i.e., by means of a function of type  (or, in HA jargon, ) where  and  are type variables which are restricted to belong to the class  and represent the row and column indices, respectively. This trick has many advantages from the formalization point of view: the type system can enforce compatible dimensions, for example, for matrix multiplication. This permits eliminating premises on the number of rows and columns: the type system will infer the dimensions for free. Throughout the paper, we will use $\alpha$ to denote the type of the elements of a matrix, whereas we will use the Latin script ( , ) when referring to the finite types of the indices of the matrices.

The HA matrix representation possesses a well-known drawback: it is cumbersome, if possible at all, to change the dimension of the matrix, or to define submatrices. Also, it seems hard to perform induction on these finite type variables. Normally some tricks are used to overcome this limitation, such as completing the submatrices with zeros and ones [1, 46], which is unsatisfactory in terms of performance and an obvious overhead during the formalization process, since this introduces a gap between the formal and the paper definitions. Despite this limitation, the HA library has been shown to be very useful for formalizing linear algebra algorithms. Indeed, based on the HA library, we successfully complete several linear algebra developments [2–4, 20].

### Another Matrix Library with Explicit Dimensions

In order to overcome the common problems with matrix dimensions when working with the HA matrix representation, Thiemann and Yamada developed a new library in a work about Jordan normal forms [53]. Their new library, from here on the *JNF* library, provides a different matrix representation, but flexible for dimensions. More concretely, a vector $(v_0, \ldots ,v_{n-1})$ is represented by a pair (*n*, *v*), where *n* is the dimension and *v* the element function (from natural numbers to the type of the elements of the vector), i.e., $v\ i=v_i$. A similar representation for matrices is provided, based on a triple (*n*, *m*, *f*) which corresponds to the number of rows, number of columns and the element function for a matrix. The type of this triple is modeled as , where $\alpha$ is the type of the elements of the matrix. They prove again many properties of linear algebra and matrices based on such a representation. Results in the JNF library require explicit conditions on dimensions and, for the moment, it is far away from having all the theorems that are formalized in the HA library, but it permits working with blocks and submatrices.

### Some Existing Classes and Types in Isabelle: mod_type, mod_ring and nontriv

In our previous works using the HA library [2–4, 20], we do not only impose finite types to model the rows and columns of matrices, but we also require more conditions (an explicit enumeration of its universe and some basic arithmetical properties) which were encoded by means of a type class named . 
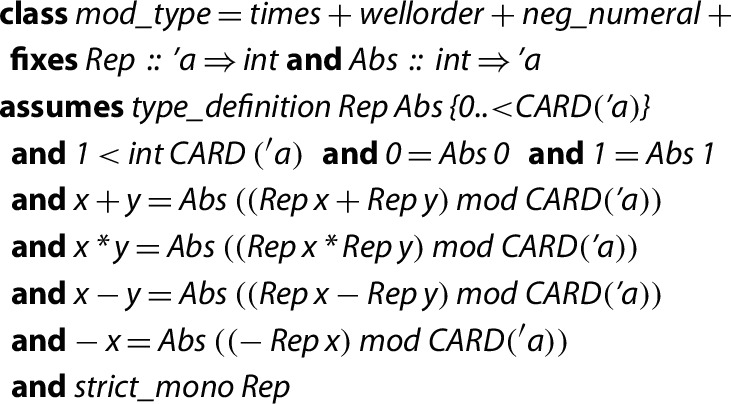
 The  class is designed to facilitate the proofs and to be later instantiated by executable types. It is necessary to define certain properties, such as imposing that every element in the diagonal of a matrix divides the next element: the finite type is not enough to impose such a condition, since there is no fixed order of its elements. In addition, the morphisms  and  are also defined within the  class: they permit the conversion between naturals and elements in a  and are accompanied by the expected simplification rules. It is important to remark that  and  do not represent an *arbitrary* bijection between  and the set of all elements of type , but  demands them to be strictly monotonic increasing functions.

Another existing concept that we will use in our work is the type , which is developed in a work on factorization of integer polynomials [23,  Sect. 3.1]. This type represents finite rings of ( ) elements. 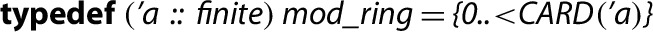 In addition, we make use of the  class, which essentially represents a finite type containing more than one element: 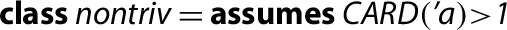


## A Connection Between the JNF Library and the HA Library with the mod_type Restriction

In a work on the Perron–Frobenius theorem [22], there has been a collaboration on the connection between the basics of the HA and the JNF libraries by means of the lifting and transfer package, whenever the indices in the HA library are modeled by finite types (class ). More concretely, that work defines functions to convert, among others, indices and matrices between both representations. For instance, a function  is defined for the indices, which is an *arbitrary* bijection between  and the set of all elements of the finite type . Analogously, its inverse function is defined as .^1^ Note that both functions have nothing to do with the morphisms  and  presented previously: the latter ones belong to the  class and they are strictly monotonic functions. Then, indices in both representations are related as follows: 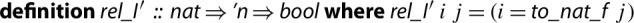 In a similar way, matrices in the JNF and the HA libraries are related: 
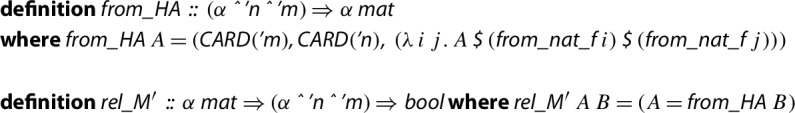
 In the definition,  is the function that converts a matrix in HA to its corresponding one in JNF,  is the access operator for matrices and vectors in HA.

Then, transfer rules like the following one are proved: 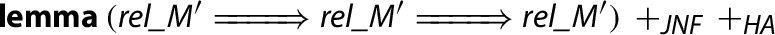 This rule means that given two matrices $A_1$ and $A_2$ in the JNF library related to the HA matrices $B_1$ and $B_2$, respectively (via ), then if matrix addition is invoked in JNF ( $A_1$ $A_2$) the result is related to the matrix addition in HA ( $B_1$ $B_2$). Transfer rules permit one to partly automate the process of sharing statements between both libraries. Putting that work together with the use of local type definitions, we can move results from one library to the other one (in a bidirectional way) if the matrix dimensions are of type  [22,  Sect. 4]. Local type definitions are the key, since they allow us to define types dynamically in proof contexts, which are mainly necessary to convert the numerical matrix dimensions in JNF into finite types in HA. More concretely, they permit one to get rid of some type class restrictions and type variables in the theorems that appear while transferring results from HA to JNF. For instance, they are used to transform statements with conditions about the  type class (such as ) to simply $i<n$ where *n* is a free variable.

However, the  class plays an important role in this work about the SNF when working with the HA library, since, similarly as in our previous works, many times the matrix dimensions cannot be modeled by (only) a finite type, but they also must satisfy additional properties. For instance, in this work we need to introduce the definition of the SNF in HA: 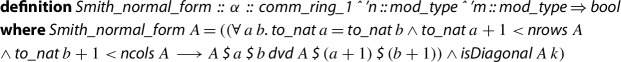 The predicate  is used to impose the matrix *A* to be diagonal. The functions  and  obtain the number of rows and columns of a matrix in HA. The input matrix *A* has type . In the definition, the quantified variables *a* and *b* are used for referring to the indices of the rows (of type  with the  restriction) and columns (of type  and with the  restriction too), respectively. Let us remark that *a* and *b* do not have the same type. This means that we cannot write  when referring to the diagonal elements. Neither we can assume $a=b$. On the contrary, we have to work with  and thus, the restriction  is added as a premise in the first condition. Let us also note that, given an index *a*, we refer to the next position as $a+1$. This is possible since the  class assumes the existence of a sum operation (class ) and some basic arithmetical properties. In addition,  demands the morphisms  and  to be strictly monotonic increasing functions. This property does not hold with , and in our case is very convenient since we can deduce useful properties, such as . Thanks to properties like this one, the  class is very useful in our previous works and, as seen above, also required in this particular development since it allows one to impose easily the divisibility condition of diagonal elements when defining the Smith normal form predicate in HA.

Thus, it is desirable to be able to move statements between both libraries, even if the matrix dimensions require the  restriction in HA. With the previously existing connection [22], this is not possible (if one tries it, one would get proof obligations like  when proving the required transfer rules for some definitions, which do not necessarily hold). To circumvent this problem, we developed a new bridge which connects the HA library with JNF, but using the morphisms  and  from the class , so that we can relate theorems and algorithms that possess such a class restriction over the indices of the matrices, and, concretely, we will be able to transfer our algorithms and results about the SNF between both representations.

We define the new relations between indices, vectors and matrices (involving ) in HA and JNF ( , , and , respectively) and prove new transfer rules for the basic operations such as matrix addition, determinants and so on.

Let us show the process to transfer statements from HA to JNF by means of a guided example. The following statement is proved in HA with the  restriction. It simply states that the first element of a square matrix in SNF divides any other element of the matrix.^2^
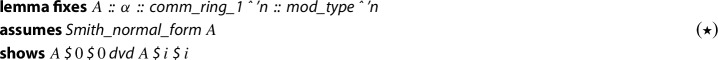


Note that we use 0 (which is of type ) to represent the first element of the type  and then,  is used to access to the first element of the matrix. However, if one only requires  instead of  for the type , we would not know if 0 is the first element of the type ; indeed, we would not know if the type  has a 0 as one of its elements.

The first step is to define the notion of SNF in the JNF library: 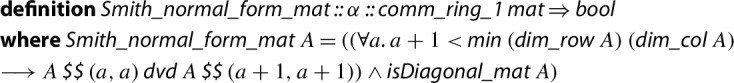


The definition is very similar to the one in HA. Here, the  symbol is used to represent the access operator for matrices in JNF and  is the predicate in JNF that requires the input matrix *A* to be a diagonal matrix.

Now, we prove transfer rules to relate the definitions involved in the statement, in this case we relate the definitions  and . 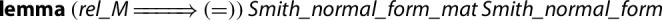


Let us recall that this lemma states that whenever a matrix *A* in JNF and a matrix *B* in HA are related (via ), then *A* and *B* are equal (related by the equality relation). The difficulty of a transfer rule depends on the number of definitions and operations that are involved as well as the similarity between the definitions in HA and JNF. In this case, the SNF definition relies on several operations and facts that also need to be related (  with ,  with ,  with , etc.). It is worth noting that  and  can be defined and related without using the  restriction, but then the corresponding transfer rule can only be proved for square matrices. Applying transfer rules to every constant presented in Lemma  we want to move from HA, we get the following statement in JNF: 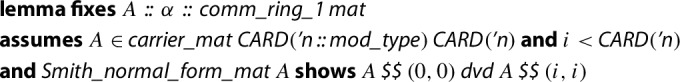


The statement assumes two new premises, namely  and  where *A* has type . Here,  is the set of all  matrices (JNF requires explicit dimensions). We would like to transform the new assumptions into *n* *n* and $i<n$, respectively, where *n* is a free variable. This can be done by means of local type definitions, since the local typedef rule [39] provides exactly what is needed: types defined dynamically in local proof contexts. However, we still would have to internalize the type class  and then abstract over its operations via dictionary construction [38]. This step is almost immediate when using the type class , because there are no operations associated with it, just the assumption , and at the end everything is reduced to assume that there exists a type (with no type class restrictions) for which there exists an isomorphism between the set of all elements of the type and $\{0,\dots ,n-1\}$, then proving that  fulfills the condition of the type class , which holds trivially, and finally,  is instantiated by .

However, as explained before,  introduces more type classes ( , , etc.) as well as associated operations and constants ( $+$, $*$, 1, etc.). Hence, the approach is more arduous, since we would have to define a predicate *S* $f'$ $g'$ ...where *S* is the carrier set and $f'$, $g'$, etc. the abstraction of all the operations and constants. This is the common approach and has been applied successfully, for instance, by Immler to connect theorems and structures of linear algebra and rings involving different representations [34]. It is feasible, but laborious. This approach would require the use of another sound extension of Isabelle’s logic by Kunĉar and Popescu: the unoverloading rule [39,  Sect. 6.2].

In our case, we take a shortcut: we use the type , which was presented in Sect. 2.5. We prove that  is an instance of the type class , that is, it satisfies all the conditions imposed by the type class: it defines a well ordering, there exists a strictly monotonic increasing function from elements of the  to the naturals, etc. Once such an instance is completed, any statement that involves a matrix of type $\alpha$     can be rewritten as $\alpha$    , where  just imposes the type to have more than one element. Fortunately, it is easy to apply local type definitions to a type , since we can rewrite  to . Then, the previous theorem can be easily transformed to: 
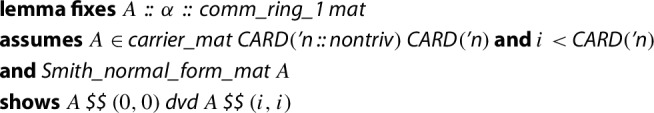


Now it is almost straightforward to substitute  by a new variable *n* using local type definitions, since it is very easy to internalize the type class  and prove that the new fresh type variable satisfies its properties (it has no associated operations or constants, so is similar to the class ). To do this, we apply the local type definition rule by creating a local type  with *n* elements and then instantiate the previous statement where . The only peculiarity which remains is that the resulting theorem restricts the new variable *n* to be greater than 1, since  demands the type  to have more than one element. This imposes the local type definition rule to be applied within a context that assumes $n>1$. Cases $n=0$ and $n=1$ have to be treated separately. They correspond to the case of matrices with no elements and with one element, respectively. In both cases, the result is trivial. Finally, we get the statement in JNF: 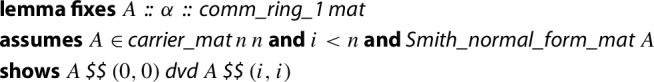


Note that, once the transfer rules are proved the rest of the process is almost immediate. As expected, the transfer rules are proved only once to relate concepts between HA and JNF, and then they are reused when moving results. The fact of being able to transfer results between both libraries by means of the connection bridges is essential and it saves much work in our development. Throughout the paper, we use both bridges continuously, not only the new connection. For instance, the following lemma was available in HA (for finite types), but not in JNF. 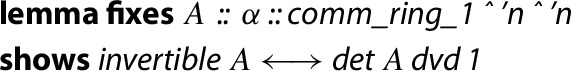


This result is very important, since it permits us to characterize the invertible matrices of any ring. We need it in the JNF library. Instead of replicating the proof, which would demand some work since it is based on several previous lemmas that are not available in JNF, we directly converted it using transfer rules and local type definitions. Once the lemma is available in JNF, we use it throughout the whole development to make the proofs easier, concretely more than 20 times. Let us remark that the connection is bidirectional and the way back from JNF to HA is easier (no local type definitions are required) once the transfer rules are provided.

As a summary of the methodology, we show here the necessary steps to move theorems back and forth between HA and JNF:

- *From HA to JNF:*
  1. define the corresponding notions in JNF in case they do not exist;
  2. prove the required transfer rules;
  3. apply the transfer rules to move the statement to JNF;
  4. deduce the result with the type class  (via the  type class) instead of having  in the statement;
  5. use local type definitions to eliminate the type variables involving matrix dimensions;
  6. for each new free variable *n* that models a matrix dimension, prove the result when $n=0$ or $n=1$ (usually trivial).
- *From JNF to HA:*
  1. define the corresponding notions in HA in case they do not exist;
  2. prove the required transfer rules;
  3. state the theorem in HA and move its assumptions to JNF by applying transfer rules;
  4. use the assumptions and the theorem proved in JNF to get the conclusion in JNF;
  5. move back the result to HA since the types of the matrix dimensions are known.

## Smith Normal Form of Diagonal Matrices

### Normal Form Computation

There are many variants of algorithms to compute a Smith normal form of a matrix, from generic algorithms that work on matrices over a PID, to more specific (and more efficient) ones in concrete structures such as the integers. Some of the generic algorithms that appear in the literature consist of two steps^3^: first a diagonal matrix is obtained and then it is transformed into Smith normal form. In this section, we present a formalization of this second step in a general setting. This step is itself interesting since there are computer programs that require it for their computations. For instance, the Kenzo system [24] uses diagonalization to compute homology, but it requires the SNF to compute homotopy (which, precisely, is computed from a diagonal matrix).

Algorithm 1 shows a way to compute a SNF *S* from a diagonal matrix *A*. It is based on the one presented by Bradley [11,  Algorithm II], but we have incorporated some basic modifications:

- The algorithm works for non-square matrices.
- The algorithm is tuned to permit singular matrices.
- The algorithm works for a matrix with coefficients in a Bézout ring, not necessarily a Euclidean domain.
- Changes have been carried out to avoid unnecessary checks on divisibility.
- We introduce how the invertible matrices *P* and *Q* (satisfying $S = PAQ$) are computed.

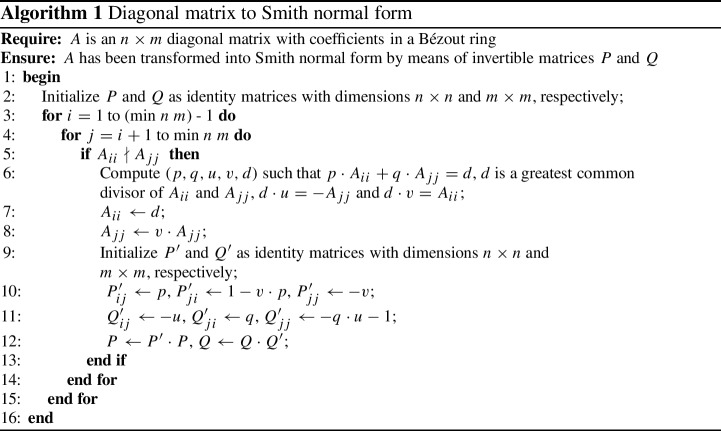


The purpose of lines 9–12 here is to compute the matrices *P* and *Q*. If these are not required, the lines can simply be dropped as they are not used elsewhere in the algorithm. Their explicit constructions are not necessary for the soundness proof of the algorithm, since one is only required to show their existence. We implemented two variants of the algorithm, one by means of a function named  which does not compute the matrices *P* and *Q*, and another slower version named  that computes them.

Let us start by showing how we implemented the first of them. Algorithm 1 is imperative pseudocode and there exist multiple ways to approach its implementation in a functional setting. In our case, we simply opted to model each loop by tail recursive functions which traverse the lists  (outer loop) and  (inner loop). The final function that transforms the diagonal matrix into Smith normal form, named , has the type: . That is, the function receives two parameters: the input matrix (with elements in a Bézout ring) and the  operation. This  operation, whose type is  (a type synonym of $\alpha \Rightarrow \alpha \Rightarrow (\alpha \times \alpha \times \alpha \times \alpha \times \alpha )$), is a function applied to two elements *a* and *b* which returns five elements (*p*, *q*, *u*, *v*, *d*) such that $pa+qb=d$, *d* is a greatest common divisor of *a* and *b*, $du=-b$ and $dv=a$; i.e., it is precisely what is needed in line 6 of Algorithm 1. It is important to note that *u* and *v* are not directly defined as $u= -b/d$ and $v = a/d$ because in abstract structures, such as Bézout rings, we do not have an explicit division operation. The  operation is a parameter of the algorithm and not a fixed operation of the type class  since this operation is not part of the structure of a Bézout ring: in general, a Bézout ring will admit many different  operations. Note that we need to use the type class  to model the indices. It is true that the use of finite types (and ) in the HA library introduces sometimes an overhead in the proofs with respect to their equivalent ones in JNF, where indices are just natural numbers, but it is, in this case, a feasible approach since we do not need to change the types of the rows and columns in our algorithm. In addition, we also provide useful results and simplification rules to minimize this overhead.

The idea of the algorithm is simple. Each step *i* consists of transforming the element $A_{ii}$ and all the elements $A_{jj}$ where $i<j$ such that $A'_{ii}$ divides all the elements $A'_{jj}$ via the inner loop by means of elementary operations and preserving the rest of elements. Iterating this procedure by all the elements in the diagonal via the outer loop, we obtain the algorithm to transform a diagonal matrix into Smith normal form.

The implementation of the version that explicitly computes the transformation matrices is rather similar:  again receives two parameters (the input diagonal matrix *A* and the  operation) and returns three matrices *P*, *S* and *Q* such that $S={PAQ}$, *S* is in Smith normal form and both *P* and *Q* are invertible. Lines 9–12 in Algorithm 1 show how the invertible matrices are constructed, but one can also view them as a composition of elementary operations applied to the identity matrix:

- $P'$ is equal to add $p \cdot \mathsf {row}\ i$ to $\mathsf {row}\ j$, then interchange $\mathsf {rows}\ i$ and *j* and add $-v \cdot \mathsf {row}\ i$ to $\mathsf {row}\ j$.
- $Q'$ is equal to add $q \cdot \mathsf {column}\ j$ to $\mathsf {column}\ i$, then add $u \cdot \mathsf {column}\ i$ to $\mathsf {column}\ j$ and finally multiply $\mathsf {column}\ j$ by $-1$.

Thus, the final matrices *P* and *Q* can be viewed as products of all $P'$ and $Q'$, or as a composition of the elementary operations presented above. Both ways to obtain them are formalized in Isabelle/HOL, so we use the most convenient one in each case during the formalization process. For example, the explicit matrices are used for efficiency to avoid repetitive elementary matrix operations and multiplication of matrices, and the view as a chain of elementary operations helps us to prove that they are indeed invertible matrices.

We sketch the soundness proof of . In each step, the invariant $S'=P' A Q'$ is preserved, where $P'$ and $Q'$ are invertible matrices and $S'$ is a matrix in SNF up to the diagonal element $S'_{ii}$, i.e., $S'$ is a diagonal matrix and each element of the diagonal up to the *i*th one divides the following one. Then, the proof consists of showing that in step *i*, the elements $S'_{ii}$ and $S'_{jj}$ where $i<j$ are modified so that the new $S'_{ii}$ divides all the new elements $S'_{jj}$, the matrix remains diagonal, the previous elements of the diagonal are not modified and they divide the new $S'_{ii}$; the whole process being performed by means of elementary operations. The proofs required a careful work due to the necessary conversions between  and natural numbers. The type of the columns can be distinct from the type of the rows, thus it is essential to use  when referring to the elements of the diagonal of a non-square matrix in the HA world. Nevertheless, thanks to the basis provided by the HA library and our previous works, we did not find hidden difficulties in the proofs during the formalization process.

We make a remark. We *hide* the induction over the indices of finite types, but instead it is carried out over the naturals and afterward the results are transformed back into their finite type version by means of the functions  and . This is possible because in each moment the finite types that model the rows and columns of the matrix are known and preserved during the whole algorithm (neither block matrices nor submatrices are required). The final theorem follows. In the statement, $$**$$ is the notation that the HA library uses for matrix multiplication. 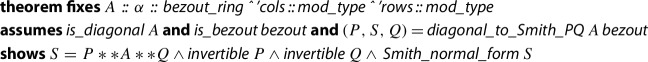


As a summary, we note some facts about the generality of this algorithm: the input matrix just requires elements in a Bézout ring (not necessarily a domain, not necessarily a PID as it is commonly defined in the literature). Second, *A* has to be diagonal but neither necessarily square nor non-singular. Third, the algorithm is parametrized by the  operation, whose existence we formally prove in a Bézout ring (but could be neither unique nor algorithmically computable). The predicate  just requires the parameter  to be indeed a  operation. This way, we have formalized an algorithm to transform a diagonal matrix into Smith normal form. This procedure will be executable as long as we provide a computable  operation. This is always possible over Euclidean domains thanks to the Euclidean division algorithm, but it is sometimes also possible in more general structures. For instance, the ring ${\mathbb {Z}}[\xi ]$, where $\xi = \frac{1+\sqrt{-19}}{2}$ is not a Euclidean domain but a PID, and an alternative method to compute the Bézout coefficients without using the Euclidean algorithm can be provided, at least in some cases, and thus a SNF can be computed [49].

### Existence criterion

Here, we show that a SNF may not exist for all diagonal matrices if one assumes a more general underlying structure than Bézout rings. We first show an alternative definition for Bézout rings which will be useful afterward.

#### Theorem 1

A ring ${\mathcal {R}}$ is a Bézout ring $\Longleftrightarrow$ all finitely generated ideals of ${\mathcal {R}}$ are principal.

This is a well-known result and our proof in Isabelle/HOL essentially follows the one presented in [12,  Theorem 6–3], with slight modifications to ease the formalization. It required about 400 lines, due to a missing property: let $\mathcal {R'}$ be a Bézout ring, then for any elements $x_1,\dots , x_n \in \mathcal {R'}$ there exist elements $\alpha _1,\dots , \alpha _n \in \mathcal {R'}$ such that $\sum _{i=1}^n \alpha _i x_i = d$, where *d* is a greatest common divisor of $x_1,\dots ,x_n$. That is, the Bézout coefficients can be obtained for a set of *n* elements of the ring, not only for every pair. The statement in Isabelle/HOL of Theorem 1 follows. 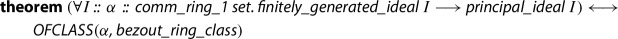


Now we are ready to prove the following theorem.

#### Theorem 2

The following three statements are equivalent:

(A) ${\mathcal {R}}$ is a Bézout ring.
(B) All diagonal matrices with coefficients in a ring ${\mathcal {R}}$ admit a SNF.
(C) All $2 \times 2$ diagonal matrices with coefficients in a ring ${\mathcal {R}}$ admit a SNF.

#### Proof

The (A) $\Longrightarrow$ (B) implication can be deduced by applying the soundness theorem of Algorithm 1, which was defined over Bézout rings in Sect. 4.1. The implication (B) $\Longrightarrow$ (C) is obvious. Now, let ${\mathcal {R}}$ be a ring. In order to prove (C) $\Longrightarrow$ (A), we must show that for all *a*, *b*, there exists some *d* such that $a{\mathcal {R}} + b{\mathcal {R}} = d{\mathcal {R}}$. Indeed, via (C) we may write

$$\begin{aligned} \begin{pmatrix}a &{} 0 \\ 0 &{} b\end{pmatrix} = P \begin{pmatrix}d &{} 0 \\ 0 &{} d'\end{pmatrix} Q \end{aligned}$$

with $d\ |\ d'$. Therefore, we find that $a, b \in d{\mathcal {R}} + d'{\mathcal {R}} = d{\mathcal {R}}$ and thus, $a{\mathcal {R}} + b{\mathcal {R}} \subseteq d{\mathcal {R}}$. For the other direction, we write

$$\begin{aligned} \begin{pmatrix}d &{} 0 \\ 0 &{} d'\end{pmatrix} = P^{-1} \begin{pmatrix}a &{} 0 \\ 0 &{} b\end{pmatrix} Q^{-1} \end{aligned}$$

to conclude $d \in a{\mathcal {R}} + b{\mathcal {R}}$ and thus, $a{\mathcal {R}} + b{\mathcal {R}} \supseteq d{\mathcal {R}}$. $\square$

To carry out the formal proof of Theorem 2 in Isabelle/HOL, it seems natural to tackle it using the HA library, since the algorithm is indeed defined in HA. The (A) $\Longrightarrow$ (B) implication is quite easy, since it just consists of using Algorithm 1 and its soundness theorem. The (B) $\Longrightarrow$ (C) implication is more problematic. We *try* to state it in the HA library as follows: 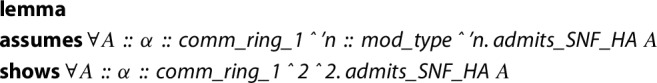


In the lemma,  is the predicate that states that the diagonal matrix *A* can be transformed by elementary operations into SNF. However, there is a problem: the type that models the dimension of the matrix is fixed (the type ). Then, we are stating the theorem for all square matrices of such a concrete dimension, and not for any square matrix. A statement like  is not valid in Isabelle/HOL, i.e., Isabelle/HOL cannot quantify over types. Thus, the HA library is not expressive enough to state the (B) $\Longrightarrow$ (C) implication of Theorem 2. The only feasible solution is to prove (B) $\Longrightarrow$ (C) (and then the complete theorem) in the JNF library, where the dimensions of matrices are modeled by natural numbers. Indeed, we can formalize it in the JNF library easily. The statement follows: 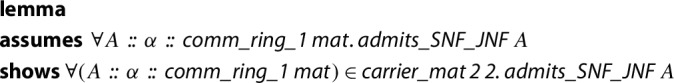


Here,  is the analogous predicate (in JNF) to . The following result is an important fact to prove the lemma. It is an alternative statement for the assumption. 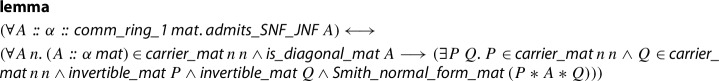


Recall that ,  and  correspond to the predicates in JNF of being invertible, diagonal and in SNF, respectively. Let us note that using this fact we now do quantify over the variable *n* (the dimension of the matrices, that is, the number of rows and columns).

The (C) $\Longrightarrow$ (A) implication is proved in JNF following the paper proof (note that this proof can also be done in HA). Now we must prove the (A) $\Longrightarrow$ (B) in JNF to have the full theorem available in the JNF library. To avoid redefining the algorithm and reproving the soundness theorem in JNF, we use the new bridge to connect HA and JNF. Following the methodology presented in Sect. 3, we get the (A) $\Longrightarrow$ (B) implication in JNF: 
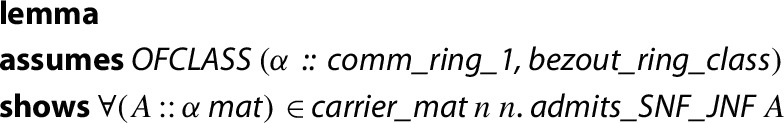


The final statement of Theorem 2 in Isabelle/HOL follows. 
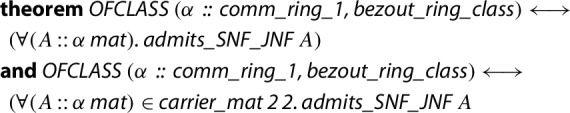


For the sake of completeness, we also include in HA the equivalence between (A) and (C) using the bridge. The sources also include a standalone (but longer) proof of (B) $\Longrightarrow$ (A) in JNF based on Theorem 1.

## Smith Normal Form of Arbitrary Matrices

### Normal Form Computation: Generic Algorithm

In Sect. 4, we presented a formalization of an algorithm to transform a diagonal matrix into Smith normal form. Here, we present a formalization of a complete algorithm to transform any matrix into Smith normal form, in a general setting.

We again parametrize the algorithm by operations: an operation to transform any $1 \times 2$ matrix into Smith normal form, likewise an operation to transform any $2 \times 2$ matrix into Smith normal form and an operation that, given an element *b* that divides another element *a*, then it returns an element *k* such that $kb = a$. As we will see, the algorithm that we will implement in Isabelle/HOL is strongly based on the use of submatrices. Thus, we make use of the JNF library.

In order to carry out the formalization, we first define a  where we fix the corresponding three operations and their properties.


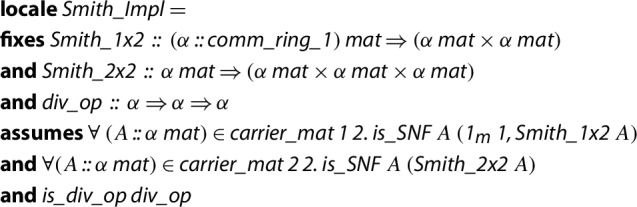


Since we are trying to work in a general setting, the type $\alpha$ represents the elements of a commutative ring with unit. We are not demanding a domain. In the code,  is a predicate that given a matrix *A* and a triple (*P*, *S*, *Q*), checks if *S* is a Smith normal form of *A* by means of the invertible matrices *P* and *Q*. 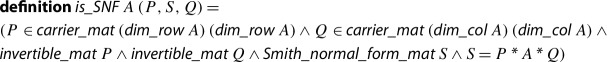,  and  are the names of the corresponding fixed operations.  is the predicate that checks if the provided division operation satisfies the required property: 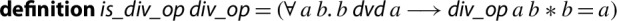


It is worth noting that, in any ring, we can always get a (non-executable) division operation using Hilbert’s choice operator. 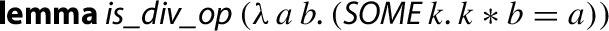


Indeed, it is possible to omit this parameter and work with Hilbert’s choice operator ( ) in the algorithm. This way, we would also be able to obtain an algorithm over generic rings. However, if one wants to execute the algorithm, one would have to provide a new algorithm in which such operation based on the  operator is substituted by an executable operation, and then demonstrate code equations between the original algorithm and the new executable one. Therefore, we considered that it was much simpler to work by means of parameters that must satisfy the required properties. Then, one would only have to instantiate the parameter and prove a single property about the operation, instead of working with code equations of the whole algorithm.

It is worth noting two facts. First, if we work in a domain (instead of a general ring), then the  operation can be removed since it can be obtained from , simply by completing the second row with zeros. Second, it is possible to work over non-commutative rings, but to do so we must fix another operation: . As we have restricted to commutative rings, this $2 \times 1$-operation can be obtained from .

In short, the locale  fixes a context where we demand that all $1 \times 2$, all $2 \times 1$ and all $2 \times 2$ matrices over a commutative ring ${\mathcal {R}}$ can be transformed into SNFs. Then, by means of an algorithm, we will prove within such a locale that all matrices over ${\mathcal {R}}$ admit the SNF transformation.

Once we have defined the locale, we can work on a generic algorithm. To do so, we will build upon a result by Kaplansky [37,  Theorem 5.1], extracting an algorithm from its proof. Based on the operations fixed by the locale, we first implement subalgorithms and prove their soundness for transforming arbitrary $1 \times n$, $n \times 1$, $2 \times n$ and $n \times 2$ matrices into Smith normal form. Then, we start with the implementation for an arbitrary $m \times n$ matrix.

The underlying idea of the algorithm follows. Given an $m \times n$ matrix *A* (with coefficients over ${\mathcal {R}}$), we first write $A_1$ for the first row and $A_2$ for the remaining $m-1$ rows.

$$\begin{aligned} A = \begin{pmatrix} A_1 \\ A_2 \end{pmatrix}. \end{aligned}$$

Then, we recurse to compute a Smith normal form of $A_2$ to obtain three matrices $P_1$, $D_1$ and $Q_1$ such that $D_1 = P_1 A_2 Q_1$, $D_1$ is a Smith normal form of $A_2$ with $P_1$ and $Q_1$ invertible matrices. Then, we have:

$$\begin{aligned} C = \begin{pmatrix} 1 &{}&{} 0 \\ 0 &{}&{} P_1 \end{pmatrix}\begin{pmatrix} A_1 \\ A_2 \end{pmatrix} Q_1 = \begin{pmatrix} A_1 Q_1 \\ D_1 \end{pmatrix}. \end{aligned}$$

Then, we define a submatrix *D* as the first two rows of *C* and define as *E* the submatrix of the remaining elements of *C*. We compute a Smith normal form of *D*, which is a $2 \times n$ matrix, such that we can get another matrix *H*:

$$\begin{aligned} H = \begin{pmatrix} P_2 &{}&{} 0 \\ 0 &{}&{} Id _{m-2} \end{pmatrix} \begin{pmatrix} D \\ E \end{pmatrix} Q_2 = \begin{pmatrix} P_2 D Q_2 \\ E Q_2 \end{pmatrix} = \begin{pmatrix} F \\ G \end{pmatrix}, \end{aligned}$$

where *F* is a Smith normal form of *D* by means of the invertible matrices $P_2$ and $Q_2$, and *G* the remaining submatrix of *H*. At this point, we let *d* be the lower left entry of *D* and *f* be the upper left entry of *F*, and prove that:

- *d* divides all elements of *E*.
- *f* divides all elements of *D*, in particular it divides *d*.
- Hence, *f* divides all elements of *E*.
- Hence, *f* divides all elements of $E Q_2$.

Thus, we have that *f* divides all elements of *H* and then *f* is used as a pivot to eliminate all the other elements in the first row and column of *H* similar to Gaussian elimination, obtaining the following matrix:

$$\begin{aligned} \begin{pmatrix} x &{}&{} 0\\ 0 &{}&{} K \end{pmatrix}. \end{aligned}$$

Repeating recursively the procedure over the $m-1 \times n-1$ matrix *K*, we get the reduction.

Our implementation in Isabelle/HOL of the algorithm follows: 
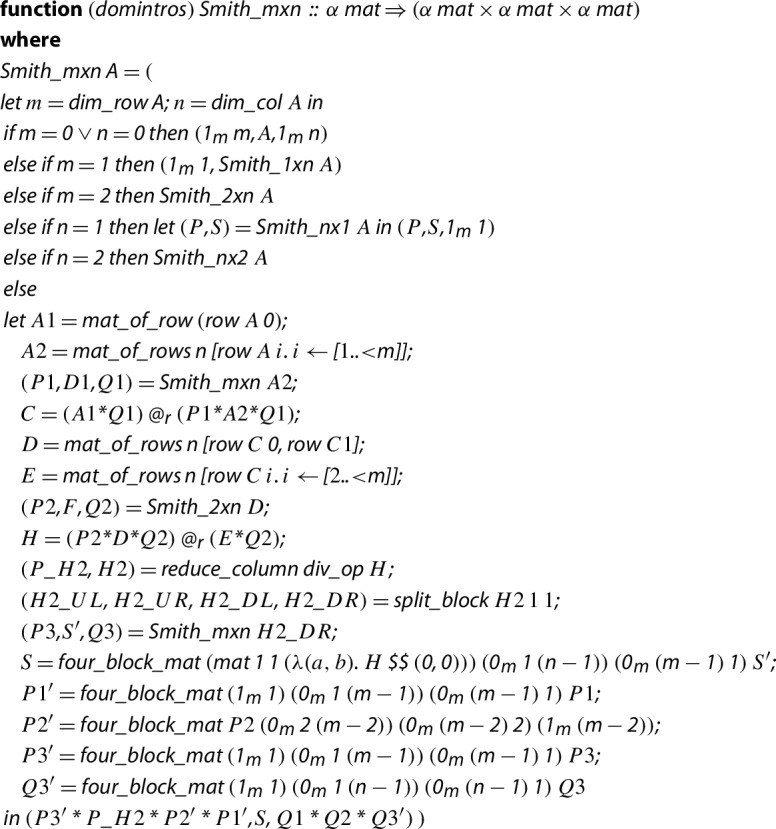


Here,  operations to build a matrix appending rows and columns, respectively.  allow us to construct the identity matrix and the zero matrix of explicit dimensions (the  stands for matrix, not for the dimension).

The paper proof of the termination of the algorithm is simple: the algorithm terminates because it has two recursive calls which involve matrices with less columns. Although our  is a total function (it is a function that works on any matrix, with no further restrictions), in Isabelle/HOL the termination proof is not so straightforward and requires some work. The reason is that the second recursive call to , i.e., the line , is carried out over a matrix that has been built involving matrices obtained from the result of the first recursive call (the line ). The problem (in other cases is an advantage) is that the JNF library requires explicit dimensions for matrices by means of natural numbers, whereas in the HA library one has them for free thanks to the type inference. That is, we have to guarantee that the dimensions of $P_1$, $D_1$ and $Q_1$ are the suitable ones in order for the remaining operations to make sense, and we cannot prove it at that point directly. To tackle this proof, we perform partial induction to get a partial correctness result about the dimensions: 
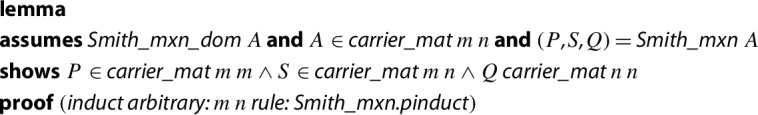


This lemma states that, given a matrix which belongs to the domain of the function  (by means of the predicate ), then the three output matrices satisfy the expected properties about their dimensions, by means of the partial induction rule . Thanks to this lemma, we prove the termination of the algorithm, since we can prove that the matrices $P_1$, $D_1$ and $Q_1$ (and the rest of matrices built from those ones) have the correct dimensions.

Once the termination is proved, we can face the soundness proof of the algorithm. We did not find hidden difficulties in the soundness proof (it can be explored in detail in the sources), but the fact of working with submatrices within a context which lacks dependent types forces us to be constantly manually proving properties about the dimensions of the involved matrices.

It is worth noting that the JNF library was extremely useful in this case. Indeed, the proof is not possible in the HA library. We have improved the JNF library with approximately 1000 lines of properties about submatrices, the operators  and , determinants and invertibility of block matrices. The final soundness theorem (within the locale ) is the following one: 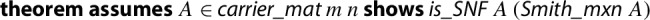


Finally, we also define the algorithm in HA based on the one in JNF. We can do it since the parameters possess fixed and known dimensions: they are operations applied to $1 \times 2$ and $2 \times 2$ matrices. Thus, we define a function  that, given a HA $m \times n$ matrix, transforms it to JNF, applies in JNF the algorithm  and finally transforms again the output to HA. Indeed, proving new transfer rules as explained in Sect. 3, we get the soundness lemma in HA.

### Normal Form Computation: Executable Algorithm

In the previous subsection we have defined a generic algorithm and proved its soundness, in both HA and JNF libraries. Now, we have to work on how to execute it. To do so, we have to provide executable parameters to the function , i.e., we have to provide executable implementations for the functions ,  and . In the case of some computable Euclidean domains (such as the integers and the ring of polynomials over a field), this is possible.

Again, we provide executable operations in both representations. Let us start with the case of the $2 \times 2$ operation. There are several approaches, one is to first diagonalize the matrix and then transform it from diagonal into SNF. To take advantage of the existing results in HA, we proceed as follows:

1. We implement an algorithm to diagonalize a $2 \times 2$ matrix in HA, taking advantage of the existing Bézout matrix in the echelon form AFP entry [3].
2. Then, we transform the diagonal matrix to Smith normal form using the algorithm proved in Sect. 4.1.
3. We define an algorithm in JNF based on the one in HA, which is possible since the types are known (type 2). Then, we transfer the results to JNF.

This way, the algorithm is defined in HA to take advantage of existing subalgorithms. The JNF version is simply a conversion to HA (which is possible since the type of the dimensions is known, since it is a $2 \times 2$ matrix), execution in HA and the output is returned to JNF.

The idea to diagonalize a $2 \times 2$ matrix is the following one. As said, we make use of the Bézout matrices defined in the echelon form development [3]. This kind of matrices allows us to reduce the upper-right and down-left elements, until they become zero. For the $2 \times 2$ case, the Bézout matrix is simple:

$$\begin{aligned} B = \begin{pmatrix} p &{}&{} q\\ u &{}&{} v \end{pmatrix}. \end{aligned}$$

Where $pA_{1,1} + qA_{2,1} = d$, *d* is a gcd of $A_{1,1}$ and $A_{2,1}$, $du= - A_{2,1}$, and $dv=A_{1,1}$. This matrix possesses good properties, such as being invertible and having determinant 1. However, the most important property is the next one:

$$\begin{aligned} B A = \begin{pmatrix} d &{}&{} *\\ 0 &{}&{} * \end{pmatrix}. \end{aligned}$$

This way, we reduce the element $A_{2,1}$. Applying similar transformations by means of the transpose of the Bézout matrix and multiplying on the right, we reduce the element $A_{1,2}$. Let us note that, when trying to get a zero in $A_{1,2}$, we probably loose the zero in $A_{2,1}$. Nevertheless, placing (via an interchange of rows or columns) the nonzero entry in the first position of the matrix, we have that the algorithm terminates, since in each step we will be reducing the entry in the position $A_{1,1}$ with respect to the Euclidean size (in the case of the ring of integers, a smaller gcd). This way, we prove (in HA) the following theorem: 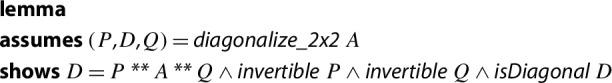


Then, we transform the algorithm from HA to JNF simply by means of the  and  operations of our bridge, since each type involved is known, and we get the corresponding lemma in JNF with the appropriate transfer rules.

Combining this algorithm to diagonalize with the one proved in Sect. 4.1 to transform a diagonal matrix into , we get the executable operation to convert any $2 \times 2$ matrix into SNF. The algorithm is available in both HA and JNF, thanks to the connection bridge.

The $1 \times 2$ case is similar to the $2 \times 2$ one. The  operation corresponds to the usual explicit division (*div*) that exists in Euclidean domains. The technical details of the implementation can be explored in the file SNF_Algorithm_Euclidean_Domain, in which is available the development required to get executable parameters for  over Euclidean domains in both libraries.

Finally, we have to instantiate the algorithm with the corresponding executable operations over Euclidean domains. Recall that the locale  fixes the three operations that are required (together with their assumptions) in JNF, and the algorithm was defined inside that locale. Locales with assumptions insert the assumptions to all facts, including the algorithms defined, when they are seen from outside the locale. This means that we have to do some work to get the code generator work on interpretations of locales. The canonical approach is to interpret the locale  globally and add new constants for all the terms we want to execute, that is, we must provide executable implementations for ,  and  over Euclidean domains at the time of interpretation. This can be done by means of the  command with dedicated rewrite definitions [27]. 
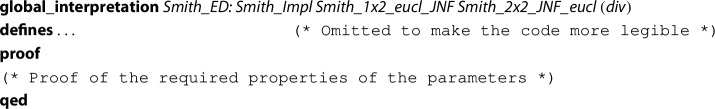


This way, we get a definition of the algorithm over Euclidean domains outside the locale , with no assumptions and executable. In fact, once we finish the global interpretation, we already have our executable algorithm over Euclidean domains in both JNF and HA, via the algorithms  and , respectively. Let us note that both libraries are collaborating in the execution: the general structure of the algorithm will be always internally executed in JNF (for the case of HA, it is converted to JNF), because submatrices are involved. However, the  operation will always execute one of its subroutines (the diagonalization step) in HA, since the Bézout matrices were implemented there. This approach makes execution rather slow, since it requires many conversions between both libraries, but it permits us to combine subalgorithms proved in different libraries. Indeed, the algorithm itself is not focused on the efficiency, but on the generality. Of course, the whole algorithm could have been implemented entirely in JNF with neither conversions to HA nor reusing its results; then, a HA-version of the algorithm and its soundness proof could be obtained again with transfer, where its execution would totally rely on the JNF library since submatrices are required.

Since our algorithm is parametrized, the performance will strongly depend on the efficiency of such parameters. For instance, in our implementation over Euclidean domains, it will depend on the efficiency on computing gcds. In computer algebra systems, specific algorithms are used for each ring to take advantage of their properties. For instance, for the integers modular arithmetic is used [51]. This permits improving the performance, but the algorithm will not be general but specific to a concrete executable ring. If we want to get efficiently a Smith normal form of some matrix in Isabelle/HOL, we can use external oracles and certify the result. This approach is explained in Sect. 6.

### Alternative Characterization of Commutative Hermite Rings and Elementary Divisor Rings

In the previous subsection we have formalized an algorithm to transform an arbitrary matrix over a ring into Smith normal form, Here, we characterize the rings where it is possible to apply a SNF algorithm for any matrix. We based our work on the well-known work by Kaplansky [37] and in particular on the generalizations presented by Gillman and Henriksen [26]. In this subsection, we intensively work with submatrices and especially we will build matrices of specific dimensions, so we will work with the JNF library. Thanks to all the work presented in the previous sections, here the formal proofs do not face major technical difficulties, only the lack of some mathematical results.

Throughout this subsection, we will work over three structures: Bézout rings, Hermite rings and elementary divisor rings. Bézout rings were already explained in Sect. 2: a Bézout ring is a ring where the Bézout identity holds and it is implemented in Isabelle/HOL in the echelon form development [3].

Hermite rings were also presented previously: they are rings where all matrices over such ring admits triangular reduction, i.e., for each matrix *A* there exists an invertible matrix *U* such that *AU* is lower triangular. The translation into Isabelle/HOL is straightforward and we use a type class to model the structure: 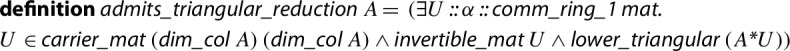

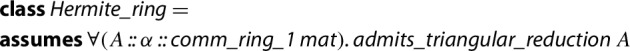


Similarly, an elementary divisor ring is a ring where all matrices can be transformed into their SNF (i.e., all matrices admit diagonal reduction). 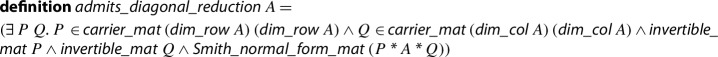

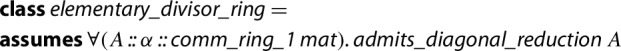


Now, the goal is to prove that all elementary divisor rings are Hermite rings, and all Hermite rings are Bézout rings. Let us start to prove that Hermite ring implies Bézout ring. To do it, we have used the alternative definition of Bézout ring: each finitely generated ideal is a principal ideal (see Definition 1). The proof took us 200 lines and the underlying idea is to fix a finitely generated ideal *I* of *n* elements, write down such *n* elements in a $1 \times n$ matrix (note that here we must use the JNF library) and then multiply it by a suitable invertible matrix *Q* to get a triangular matrix whose first element will generate the ideal *I* and thus *I* is principal. The final statement follows: 
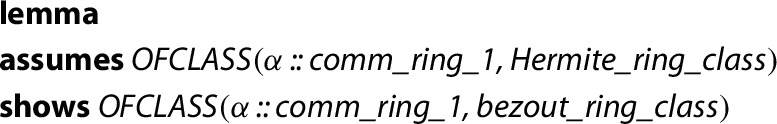


To prove that any elementary divisor ring is a Hermite ring, we first show that the operation  (that is, that we can transform any $1 \times 2$ matrix into Smith normal form) is equivalent to state that we can triangularize any matrix. We follow a proof by Kaplansky [37,  Theorem 3.5], who essentially provides an algorithm to triangularize any matrix, given an operation to triangularize any $1 \times 2$ matrix. Again, it is a proof that requires the use of submatrices and block matrices. It needed 400 lines, excluding the auxiliary lemmas about invertibility of block matrices that we proved. As a corollary, we get the remaining implication: elementary divisor ring implies Hermite ring. 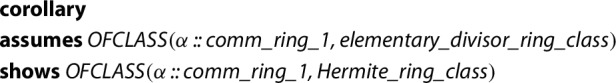


Once we have proved the chain of inclusions among the three kinds of rings, we move on to characterize Hermite rings and elementary divisor rings. We start with the following result on Hermite rings, which corresponds to [26,  Theorem 3].

#### Theorem 3

A commutative ring ${\mathcal {R}}$ with identity is an Hermite ring if and only if it satisfies the condition**T:** for all *a*, $b \in {\mathcal {R}},$ there exist $a_1,$ $b_1,$ $d \in {\mathcal {R}}$ such that $a = a_1 d,$ $b = b_1 d$ and $(a_1, b_1) = (1)$.

The notation $(a_1, b_1) = (1)$ just means that the ideal generated by the elements $a_1$ and $b_1$ is equal to the ideal generated by the unit, i.e., the whole ring ${\mathcal {R}}$. Its proof in Isabelle/HOL only required 100 lines (excluding the proofs of the necessary previous results, such as the theorem presented before that states that any matrix admits triangularization, given an operation to triangularize any $1 \times 2$ matrix) and was performed with no major difficulties, except for some missing properties about rings and ideals. Note that we can restrict ourselves and prove Theorem 3 for $1 \times 2$ matrices (which essentially follows the same proof, but with no need to show that triangularization over $1 \times 2$ matrices permits triangularization over any matrix). This result is interesting, since the proof of Hermite ring $\Longrightarrow$ Bézout ring becomes straightforward (with no use of Theorem 1). This alternative proof is also formalized in the sources.

With respect to the characterization of elementary divisor rings, the final goal is to prove the following theorem.

#### Theorem 4

A commutative ring ${\mathcal {R}}$ with identity is an elementary divisor ring if and only if it is an Hermite ring that satisfies the condition**D:** for all *a*,  *b*, $c \in {\mathcal {R}}$ with $(a,b,c) = (1),$ there exist *p*, $q \in {\mathcal {R}}$ such that $(pa,pb+qc)=(1)$. Thus, ${\mathcal {R}}$ is an elementary divisor ring if and only if it satisfies **T** and **D**.

Such a theorem is a generalization of the rather restrictive version presented by Kaplansky [37,  Theorem 5.2]. Also the proof of the theorem is based on several previous results [26], apart from Kaplansky’s work.

This proof required 500 lines, excluding some missing auxiliary lemmas, whereas in the original paper proof [26] it requires about 30, including the reference to previous results [37]. The proof uses properties already explained, such as the characterization of Hermite rings and that Hermite ring implies Bézout ring. It also uses the algorithm presented in the previous subsection, since the proof defines functions to transform $1 \times 2$ and $2 \times 2$ matrices into Smith from the operations T and D. Then, the parametrized algorithm presented in the previous subsection is applied. 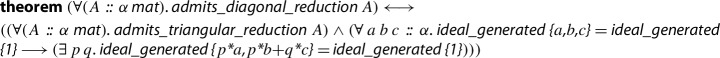


This way, it is always possible to obtain the functions to transform $1 \times 2$ and $2 \times 2$ matrices into SNF, which are necessary for the algorithm presented in the previous subsection to work, from the operations T and D.

## Result Certification Approach

In the previous section, we have presented a general verified algorithm to transform arbitrary matrices into their SNFs. If one is simply interested in the result of computing a Smith normal form over a concrete structure, such as integer matrices, a result certification approach is a valid alternative. The approach is well known: compute externally and check the output in a theorem prover. In our case, the external algorithm (efficient oracle) must provide five matrices: *P*, *S*, *Q* and also the inverses $P^{-1}$ and $Q^{-1}$. We could really work with only the matrices *P*, *S* and *Q*, but then we would have to check whether *P* and *Q* are indeed invertible matrices within Isabelle/HOL, which would affect the performance. This way, given $P^{-1}$ and $Q^{-1}$, only a matrix multiplication is required to check each one. Since the main reason to use the certified approach is the performance, the checker is implemented in JNF to make use of the Strassen matrix multiplication algorithm (which requires block matrices). The final soundness statement follows: 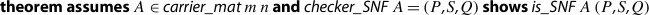


This approach permits us to solve the problems with the performance of our verified version. Moreover, both approaches are compatible: the verified approach provides a formal proof of an algorithm over general structures, whereas the certified approach provides a connection to external efficient algorithms, whose output is verified within Isabelle/HOL. The performance of the certified approach strongly depends on the performance of the external algorithm: since the properties validated by the checker are inexpensive, the checker does not cause a huge overhead. However, the need of the $P^{-1}$ and $Q^{-1}$ matrices slows down the external algorithm, but in any case it is faster than checking invertibility within Isabelle/HOL.

## Uniqueness of the Smith Normal Form

The SNF is unique up to multiplication by units. This statement can be expressed in both the HA and JNF libraries. However, the proof requires an intensive use of submatrices and minors (determinants of submatrices). Since submatrices are a delicate issue in the HA library, the appropriate approach is to state the theorem in HA, but prove it in the JNF library. Indeed, a formal proof in the HA library is not directly possible, since induction on the dimension of submatrices is required. We first show the definition of submatrix in JNF, which was introduced by Bentkamp. 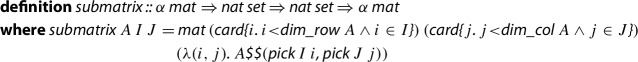


Here, *I* and *J* are the subsets of ${\mathbb {N}}$ which restrict the indices of *A* that define the elements of the submatrix. The function  is used to relate the indices of the input matrix to the ones of the submatrix, i.e., *I* *i* is the *i*th largest element of *I*. This way, the element ( *A* *I* *J*)  (*i*, *j*) is the one in the position ( *I* *i*, *J* *j*) of the input matrix *A*. Note that if $I=\mathsf {UNIV}$ (where $\mathsf {UNIV}$ represents the set of all elements of type ), then *I* $i = i$, i.e., we choose all rows (analogous with respect to *J* and the columns).

Now, we sketch our proof of the uniqueness of the SNF of a matrix, which is an adaptation of the one presented in [32,  p. 76]. The first step consists in showing that any minor of order *k* (the determinant of a submatrix with *k* rows and *k* columns) of a diagonal matrix is either 0 or the product of *k* elements of the diagonal (up to sign). The proof is carried out by induction on *k*. Although our proof in Isabelle/HOL required some work due to some missing properties about submatrices, the JNF library and its submatrix representation were very useful and allowed us to formalize the result.

Then, the following equivalences are the key to prove the uniqueness:

$$\begin{aligned} \prod _{i=1}^k d_i {{\mathop {\equiv }\limits ^{(1)}}} \gcd (\bar{S}_{k}) {{\mathop {\equiv }\limits ^{(2)}}} \gcd (\bar{A}_{k}). \end{aligned}$$

In the formula, $d_i$ is the *i*th element of the matrix *S*, which is a Smith normal form of a matrix *A*. $\bar{A}_{k}$ represents the set of all minors of order *k* of *A* (analogous for $\bar{S}_{k}$). Thus, we are stating that the product of the *k* first elements of *S* is associated, i.e., equal up to multiplication by units, to the $\gcd$ of all minors of order *k* of *S* and also to the $\gcd$ of all minors of order *k* of the original matrix *A*.

In order to prove (1), we proceed as follows. First, we show that the left-hand side divides the right-hand side. Let *b* be a nonzero minor of order *k* of *S* (if the minor is 0, we are done). Since *S* is in SNF, then it is diagonal and thus *b* can be written as the product of *k* elements of the diagonal of *S*. Let *J* be the (ordered) list of those *k* elements, then each $d_i$ divides the *i*th element of *J*, and this implies the result. The other direction is actually quite easy, indeed $\prod _{i=1}^k d_i$ is a minor of order *k* of *S* and then the $\gcd$ of all minors must divide it.

Before proving (2), we need an essential result: the Cauchy–Binet formula. This formula relates the determinant of the product of rectangular matrices to a summation of determinants of submatrices.

### Theorem 5

(Cauchy–Binet formula) Let *A* be an $$n\times m$$ matrix and *B* an $$m \times n$$ matrix. Let $${\mathcal {C}}$$ be the set of *n*-combinations of $$\{1,\dots ,m\}$$ (the set of the subsets of $$\{1,\dots ,m\}$$ with cardinality *n*). Then, the Cauchy–Binet formula states that $$\mathsf {det} (AB) = \sum _{I\in {\mathcal {C}}} \mathsf {det} (A_I) \cdot \mathsf {det} (B_I)$$, where $$A_I$$ (resp. $$B_I)$$ is the submatrix of the *n* columns (resp. rows) with indices in *I*.

Our proof in Isabelle/HOL of this fact follows the argument presented in [54]. Nevertheless, we substantially modified some parts of it [concretely, while proving Eq. (11) of that article] to base the reasoning on properties of determinants that were already proved in JNF. The final statement in Isabelle/HOL follows: 
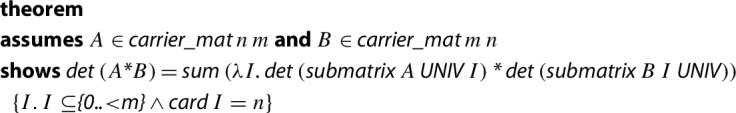


Once the Cauchy–Binet formula is formalized, we can tackle the proof of (2). The key is to prove the following rewrite rule for all matrices *P*, *A* and *Q*, sets of indices *I* and *J* of cardinality *k*, and sets ${\mathcal {I}} = \{I'.\, I'\subseteq \{0..<n\} \wedge \mathsf {card}\, I'=k\}$ and ${\mathcal {J}} = \{J'.\, J'\subseteq \{0..<m\} \wedge \mathsf {card}\ J'=k\}$.

$$\begin{aligned}&\mathsf {det}(\mathsf {submatrix}\ (PAQ)\ I\ J) =\\&\sum _{ I' \in {\mathcal {I}}} \sum _{ J' \in {\mathcal {J}}} \mathsf {det}\, (\mathsf {submatrix}\ P\ I\ J') \cdot \mathsf {det}\, (\mathsf {submatrix}\ A\ J'\ I') \cdot \mathsf {det}\, (\mathsf {submatrix}\ Q\ I'\ J). \end{aligned}$$

The proof of this fact is performed using the Cauchy–Binet formula and properties of multiplication and composition of submatrices. In (2), to prove that the right-hand side divides the left-hand side, one just has to note that $\gcd (\bar{A}_{k})$ divides each element of such a summation (concretely, each $\mathsf {det(submatrix}\ A\ J'\ I')$), and then we get the result. The reasoning for proving the other way consists of applying the same rewrite rule again, but taking into account that *P* and *Q* are indeed invertible matrices.

Putting everything together, we get the statement for the uniqueness, since given two matrices *S* and $S'$ which are Smith normal forms of a matrix *A*, then $S_{ii}$ and $S'_{ii}$ are associated. The final statement (in JNF) is as follows: 
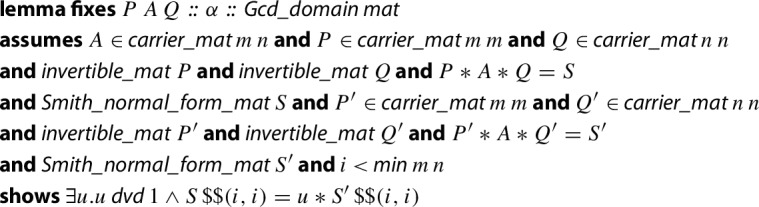


Nevertheless, our algorithm was defined in the HA library where also its soundness theorem is proved, so we transfer the result from JNF to HA following the approach presented in Sect. 3. Let us note that the uniqueness is proved on a GCD domain (a more general structure than Bézout domain). We also get the equality of the matrices *S* and $S'$ (in both HA and JNF) once the definition of the SNF is parametrized by a *complete set of non-associates*, i.e., a set of elements of ${\mathcal {R}}$, one from each equivalence class defined by the equivalence relation of being associate elements [51].

In this concrete case we can also transfer an intermediate result to HA: the Cauchy–Binet formula. Although this formula involves submatrices, in this concrete case something special happens: we do know the type of each submatrix involved in the formula in the corresponding HA statement. If the original matrices are of type  and , respectively, then the submatrices will have type . Thus, in this case we can define submatrices in HA. 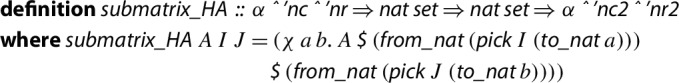


Here, $\chi$ defines a lambda-expression when working with the vector and matrix representation presented in HA.

By means of transfer rules we can relate submatrices in HA to submatrices in JNF, but in a context assuming that the cardinality of the sets *I* and *J* that define the submatrices are equal to the cardinality of the new types of the submatrices. 
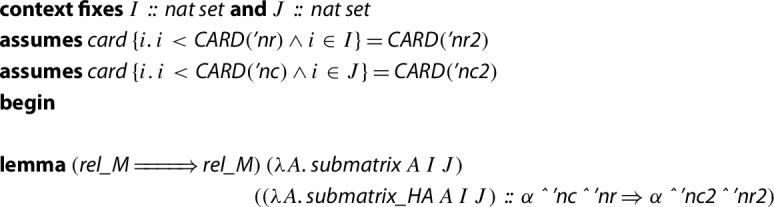


Now, using this new transfer rule outside the context properly, we can move the result from JNF to HA. Then, we obtain the following statement: 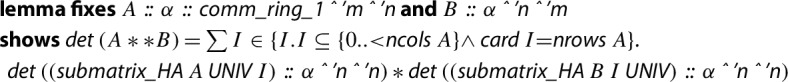


Note that the type of the submatrices must be explicitly provided, and this is possible thanks to the fact that the type variable  is already fixed.

## Related Work

There are linear algebra developments in most theorem provers [15, 16, 25, 44, 47], above all focused on vector spaces properties. However, only Coq provides a formal correctness proof of the SNF thanks to a development by Cano et al. [13], which is the closest work to ours. Such a development consists of approximately 9000 lines of code^4^ and uses the SSReflect proof language. This work differs in the sense that the authors provide formal proofs of facts that are not covered in our work, for instance, the classification theorem for finitely presented modules. They provide three formalizations of the Smith normal form algorithm: over a Euclidean domain, over a structure that they call constructive principal ideal domain (a Bézout domain with a well-founded divisibility relation) and over an integral domain with a division operator and a $2 \times 2$ Smith operation, which is actually the closest to what is presented in this paper.

The Coq development makes strong use of dependent types; indeed, matrices are implemented as finite functions over finite sets of indices where dependent types are used to ensure well formedness. This makes it easier to work with submatrices and permits induction over the dimension, which are problematic issues in the HA library. Since Isabelle/HOL lacks dependent types, we had to switch conveniently between two representations (the HA and JNF libraries) using the lifting and transfer package and local types definitions to get the results. Dependent types also facilitate some definitions and proofs, for example, the authors of [13] also use dependent types for defining the set ${\mathcal {Z}}_{nm}$ of functions from $\{0,\dots ,n-1\}$ to $\{0,\dots ,m-1\}$ where *n* and *m* are parameters. This set is necessary at some point for proving the Cauchy–Binet formula. As a definition like that one cannot be precisely cast in the HOL type system and in fact functions in HOL are total, we usually have to somehow complete the functions of such a set for values outside the domain. Such functions must have type  in Isabelle/HOL; whereas in Coq they can write $\{\texttt {ffun}\,\texttt {'I}\_\texttt {n}\,\rightarrow \,\texttt {'I}\_\texttt {m}\}$ in order to implement the type of functions with a finite domain (’I_n and ’I_m represent the types with *n* and *m* elements, respectively). In this concrete example, we model the set ${\mathcal {Z}}_{nm}$ as a function in Isabelle/HOL that receives two parameters (*n* and *m*) and returns the set of functions which map values in $\{0,\dots ,n-1\}$ to $\{0,\dots ,m-1\}$, with the extra condition that each $f \in {\mathcal {Z}}_{nm}$ satisfies $\forall i.\ i \notin \{0,\dots ,n-1\} \longrightarrow f\ i = i$. With this trick, we can ensure the injectivity of each $f \in {\mathcal {Z}}_{nm}$ provided that *f* is injective over the set $\{0,\dots ,n-1\}$, i.e., we usually have to *complete* the functions outside the desired domain to preserve useful properties in each case. Otherwise, too many cases would arise in the proofs and this could even break them, for example, when two functions of ${\mathcal {Z}}_{nm}$ must be composed. This approach clearly introduces an overhead in the formalization process in Isabelle/HOL, where functions must be total, compared to the same proofs with dependent types; but it is a workable solution (and it seems there is no better alternative). Another example where the use of dependent types makes this kind of formalization easier is in the termination proof of the algorithm presented in Sect. 5.1. As explained there, since our matrices must be accompanied with their explicit dimensions (modeled with natural numbers), we have to prove that the involved matrices possess the correct dimensions, which required a partial induction. However, in a setting with dependent types, one would have this almost for free.

One difference with respect to the development in Coq is that we allow zero divisors (we do not assume that the ring is a domain). The correctness proofs of the algorithms essentially follow the same reasoning as in a domain and indeed the difficulty is similar, but the proofs become longer (we estimate a 10%) since we need to treat more cases. The Coq development includes a characterization of elementary divisor domains^5^ based on the Kaplansky condition presented in [37,  Theorem 5.2]. In our case, we provided a characterization of elementary divisor rings (not restricted to domains) in Theorem 4. To this end, we had to formalize most of the results presented in an article by Gillman and Henriksen [26], which requires more effort but generalizes the statement of the Coq development. The main contribution compared to the Coq development is that ours has been carried out in HOL, a context without dependent types, making use of local type definitions and a new bridge to switch between two representations. We sketched this idea at a workshop [21].

Local type definitions are still considered as an experimental extension of higher-order logic and there are very few developments that have used it. As far as we know, apart from the basic examples provided by the implementation of the rule and the work presented in this paper, there only exist four developments that have used local type definitions. The first one is the library about the Perron–Frobenius theorem [22], which has already been mentioned in Sect. 3. The second one presents a formal correctness proof of the Berlekamp–Zassenhaus algorithm [23], an algorithm to factor polynomials. There, local type definitions are used to connect three different representations of finite rings. This library has also been mentioned in Sect. 3 since we make use of the type  implemented in it. The authors of this paper collaborated in both the Perron–Frobenius and the Berlekamp–Zassenhaus development. The last development is a work that defines and proves basic properties of smooth manifolds [35], where the authors transform an existing (type based) library of linear algebra (which includes definitions and results on groups, rings, vector spaces...) into one with explicit carrier sets. There also exists an interesting example on how to use local type definitions to move Cramer’s lemma to the JNF library [10,  Sect. 6].

Theorem 2 relates the existence of a Smith normal form to the properties of the underlying structure. It is also known that, if all matrices over a commutative ring with unit ${\mathcal {R}}$ have a SNF, then it does not follow that ${\mathcal {R}}$ is a principal ideal domain [40]. Moreover, there are examples of Bézout rings that are not Hermite rings and also examples of Hermite rings that are not elementary divisor rings [31]. However, it is a well-known open problem to decide whether a Bézout domain is always an elementary divisor domain [30].

## Conclusions and Future Work

In this work, we have formalized several facts related to the Smith normal form of a matrix. An important contribution of this work is its methodology of formalization: since many results and algorithms about the SNF require the manipulation of submatrices, we had to switch between two existing libraries (HA and JNF). Indeed, there are proofs that cannot be performed in the HA library and even theorems that cannot be stated there, since quantification over type variables is not possible in Isabelle/HOL. Sometimes one prefers proving a theorem in one of the libraries because its proof is easier. It also happens that some of the required results exist in the HA library but not in the JNF one. To easily switch between both libraries, we use bridges to connect them and move results bidirectionally using the lifting and transfer package and local type definitions. This way, both libraries are combined to provide the advantages of a type based formalization (in which sometimes proofs are more concise and direct since the dimensions are ensured via type checking) with the flexibility of explicit dimensions as natural numbers, all of this in Isabelle/HOL with its powerful proof automation tools. This permits working with simple type theory in a context (matrices, submatrices, blocks, etc.) that is usually considered more adequate for dependent type theory. Following this methodology, we have formalized an algorithm that transforms a diagonal matrix into Smith normal form, which is sometimes the second step to obtain a general algorithm that works for any matrix. This verified algorithm accomplishes the task in a general setting: it works for any matrix, including non-square and singular ones, and it is defined for matrices with coefficients in Bézout rings. Indeed, we have presented a proof of Theorem 2, which shows that there is no more abstract structure where the desired transformation can be carried out for all matrices. A complete Smith normal form algorithm which works for any matrix over elementary divisor rings is also provided, also in a general setting via parametrization by functions. To provide an efficient alternative to the verified algorithm, we also formalized a result certification approach. In addition, we have formalized characterizations of Hermite rings and elementary divisor rings, following the generalizations presented in [26] and a formal proof on the uniqueness of the SNF.

Some parts of the development were quick to develop and are very readable, since the code includes comments and examples. On the other hand, the development of the infrastructure to connect both libraries (HA and JNF) and sometimes the use of it together with local type definitions are more technical. But once the basics of the bridge are developed, one just has to connect the definitions of the different representations, which is usually an easy transfer lemma. The proof engineering methodology presented in this paper is scalable and replicable in other developments. It extends the previous connection by allowing statements with the  restriction, i.e., it permits easily transferring a lemma that requires HA and  to JNF and vice versa, and one can also combine algorithms in different representations. This shows that the combination of the lifting and transfer package with local type definitions is a workable strategy to share theorems between different representations, and it even makes it easier to work in a setting with no dependent types. In fact, the new bridge has already been successfully used in a development about modular algorithms for computing reduced lattice bases and Hermite normal forms [9]. Such a development required to move almost all final theorems of the echelon form and Hermite normal form AFP entries [18, 19] (which are large developments; around 8000 and 2300 lines of code, respectively), from HA to JNF. The JNF library is growing and is a nice candidate to substitute the HA representation in the long term due to the troubles when reasoning with matrix dimensions. Indeed, according to the AFP statistics, it is one of the most used entries. However, it is usual that some entries that use the JNF library again prove theorems that are already part of HA. For instance, the Gröbner basis entry [43] proves from scratch theorems about row spaces (such as the row space of $P\cdot A$ is equal to the row space of *A* if *P* is an invertible matrix) that were already proved in HA with the  restriction. The use of the new bridge would benefit the works that rely on JNF.

## Footnotes

1 In Isabelle/HOL, these functions are named  and , respectively.

2 This theorem is rather simple and can be proved in JNF by induction in very few lines. In fact, both libraries include the result (for arbitrary matrices, not necessarily square) without using the bridge. For the sake of simplicity, we present it here proved using the bridge to show a guided example of its use.

3 Sometimes both steps are merged and the transformations are performed at the same time.

4 According to the companion material of the paper [13] presented in https://perso.crans.org/cohen/work/edr/.

5 In their paper [13], they call them elementary divisor rings, but they assume that the ring is an integral domain.
